# Metabolic syndrome in lupus patients in northeast of Iran, and their lifestyle habits.

**Published:** 2016

**Authors:** Mohammad Reza Hatef-Fard, Mina Khodabandeh, Maryam Sahebari, Majid Ghayour-Mobarhan, Zahra Rezaieyazdi

**Affiliations:** 1 Rheumatic Diseases Research Center, School of Medicine, Mashhad University of Medical Sciences, Mashhad, Iran.; 2 School of Medicine, Mashhad University of Medical Sciences, Mashhad, Iran.; 3 Biochemistry and Nutrition Research Center, School of Medicine, Mashhad University of Medical Sciences, Mashhad, Iran.

**Keywords:** Systemic lupus erythematosus, SLE, Lupus, metabolic syndrome, Diet, Exercise, Life style

## Abstract

**Background:**

Systemic lupus erythematous is an autoimmune disease associated with atherosclerotic manifestations or metabolic disturbance due to inflammation. The aim of this study was to determine frequency of metabolic syndrome (MetS) in SLE compared to healthy controls.

**Methods:**

In this cross-sectional study, 150 SLE patients and 220 healthy volunteers were enrolled. MetS was diagnosed according to ATPIII criteria. Patients and controls were compared according to prevalence of MetS. In addition, SLE patients with and without MetS were compared according to laboratory parameters. Each patient also fulfilled a checklist about routine daily activities and diet program. Data were analyzed by SPSS-11 software.

**Results:**

MetS was significantly lower in SLE than healthy controls (18% vs 29.1%, P=0.015). Disease manifestations, major organ involvement, serum values of complements and anti-DNA antibody and pharmacological therapy did not correlate with MetS occurrence in patients. The mean TG, FBS, systolic and diastolic BP were statistically higher in lupus patients compared to healthy volunteers in contrast to waist circumference. HDL-cholesterol serum values did not show any significant difference between two groups.

**Conclusion:**

It seems that despite higher values of blood pressure, serum lipids and glucose in lupus patients, the cumulative metabolic components were in a manner to make MetS more prevalent in healthy volunteers. As far as life habits are concerned, lupus patients in general did not exercise enough and did not go on a healthy diet despite of glucocorticoid therapy and hypertension.

Systemic lupus erythematous(SLE) is an autoimmune disease in which breakdown of self-tolerance leads to out of control activity of innate and adaptive immunity and multi-organ damage (1). Endothelial dysfunction, inflammatory cytokines, excess lipid production, protein loss, oxidative stress, hypercoagulable state, and some medications precede imbalance in lipid profiles in favor of atherosclerosis, which is supposed to be the leading cause of morbidity in lupus (2). Metabolic syndrome (MetS), seems to be more prevalent in lupus patients compared to healthy people in some populations (2-11). MetS in lupus is not a very new research area; but several studies in different populations have been conducted with this purpose yielding paradoxical results mainly related to genetic variety, lifestyle habits such as diet and physical activities, age, diagnostic criteria and economic variables (3, 12-18).

In rheumatoid arthritis which is an inflammatory disease similar to SLE, we have found lower prevalence of MetS compared with age and sex-matched healthy controls in our region (19). Accelerated atherosclerosis is a well-known common social health issue in the general population as well as in inflammatory diseases. Nonetheless, MetS in the geographic region of this study may not be a good indicator of coronary ischemic disease (20). Additionally, there are pros and cons for the exact validity of MetS predictive value for cardiac accidents (21). The aim of this study was to determine the prevalence of MetS in SLE patients versus healthy controls with regard to lifestyle habits of these patients to find out whether there is a correlation between MetS and disease manifestations or not. Moreover, a small interview about the lifestyle habits of those patients was conducted.

## Methods

Patients and control: In this cross-sectional study, 370 participants consisted of 150 patients who fulfilled the ACR criteria for SLE (22) and 220 healthy volunteers were enrolled. SLE patients were selected from a cohort of MetS surveillance (23) presented for follow-up to Rheumatology Clinic of Mashhad University of Medical Sciences, Iran. Serum values of anti-DNA antibody, complements, proteinuria and history of organ damage secondary to lupus were also considered for all patients. 

At the same time, data regarding demographic, anthropometric, clinical, and laboratory features were collected for the healthy controls. MetS was defined by the presence of any three of the following five parameters according to the “National Cholesterol Education Program's Adult Treatment Panel III report” or ATPIII: abdominal obesity based on waist circumference (>102 cm in men and >88 cm in women), triglycerides at least 150 mg/dl, high-density lipoprotein (HDL) below 40 mg/dl for men and 50 mg/dl for women, blood pressure at least 130/85 mm Hg, and fasting glucose at least 110 mg/ dl (or those who were under treatment for hyperlipidemia , hypertension, or diabetes) (24). All participants signed an informed written consent prior to study enrolment; Ethics Committee of Mashhad University of Medical Sciences approved this study.

**Anthropometric measurements:** Anthropometric parameters including height, weight, waist circumference, and body mass index (BMI), as well as diastolic and systolic blood pressure were measured for all participants. Height (centimeters) was recorded in all participants without shoes, and weight (kilograms) was measured for participants in light clothing using electronic weighing scales. Waist circumference (WC) was determined at the level of the umbilicus) and hip circumference (defined as the widest part of the body below the waist) measurements were also taken, and the WHR was calculated by dividing hip circumference to WC and BMI was calculated by dividing weight in kilograms divided by the square of the height in meter. For measuring blood pressure, the participants remained seated for 15 min and at least two readings of blood pressure were taken.

We also filled out a simple checklist about duration and severity of physical activity of each patient in a day. If patients had more than an hour/day physical activities for their job or more than 30 minute/day exercise program, they were classified as highly active. We additionally asked about supplement therapy and low salt and low fat dietary program in patients, especially those treated with glucocorticoids.

Statistical analysis: The SPSS software (Version 11.5, Chicago, IL, USA) was used for statistical analysis. Kolomogrov–Smirnov test was used to evaluate the normality of data. Values were expressed as mean±SD for normally distributed variables and median with inter-quartile range (IQR) for non-normally distributed data. Baseline demographics and clinical characteristics were compared between groups using independent samples t -test, Mann–Whitney U test, chi-square, and/ or Fisher's exact test, were used when appropriate. Bivariate correlation was assessed using Pearson's and Spearman's correlation coefficients for normally and non-normally distributed data respectively. Anti-DNA antibody / lab-reference ratio referred to as anti-DNA/R was calculated for measurement of elevated anti-DNA serum values. A p-value <0.05 was considered significant.

## Results

**Demographics:** Important demographics and anthropometric measurements of the patients and controls are presented in table 1. 

**Lupus manifestations:** The mean duration of disease was 42.7±60.6 months. Among patients, 24.6% had proteinuria or renal involvement, 2.7% showed central nervous system involvement, 3% had pericardial effusion. One hundred and twenty four of the patients were treated with prednisolone. The average cumulative dose of corticosteroid therapy after disease development considering glucocorticoid pulse therapy was 37.1±47.08 mg/month. 79.8% of the patients received hydroxychloroquine at dosage of 6 mg/kg/day. Twenty seven percent of them received cytotoxic drugs. The mean C3 level was 98.18±39.06 mg/dl, the mean C4 level was 21.95±13.34 mg/dl and the mean anti-DNA/R was 4.4±14.5. 

**Table 1:** Demographics, laboratory and anthropometric data of patients and controls, comparing them after adjustment for age (co-variance analysis)

**Demographics, laboratory and** **anthropometric data**	**Lupus patients**	**Healthy controls**	**P-value** **between groups**
MetSn(%)	27 (18%)	64 (29.1%)	0.015 (x^2^=5.9)
TG (mg/dl)	126.18 (±32.5)	116.02 (±10.4)	0.016 (z=2.4)
HDL (mg/dl)	44.09 (±5.04)	44.9 (±1.5)	0.07 (t=1.8)
LDL(mg/dl)	96.27 (±16.2)	109.32 (±3.11)	<0.001 (t=8.9)
FBS (mg/dl)	94.31 (±22.3)	80.63 (±2.5)	<0.001 (z=15.6)
Waist circumference (cm)	86.7 (±6.8)	90.2 (±2)	<0.001 (t=6.03)
Hip circumference (cm)	95.93 (±8.3)	104.2 (±9.1)	<0.001 (t=8.8)
Waist/hip circumference (cm)	0.9 (±0.08)	0.87 (±0.07)	<0.001 (t=4.2)
Body mass Index (BMI) (kg/m^2^)	24.56(±2.4)	27.8(±0.78)	<0.001 (z=13.5)
sysBP (mm/h)	115.4 (±7.4)	111.3 (±4.2)	<0.001 (z=8.7)
dysBP (mm/h)	76.4 (±5.5)	73.8 (±3.3)	<0.001 (z=6.7)
Age (year)	30.05 (±10.1)	34.7 (±0.7)	0<001 (z=9.2)
Sex =female n(%)	136 (90.7%)	200 (90.9%)	0.92 (x^2^=0.006)

MetS: Metabolic Syndrome; TG: triglyceride; HDL: high density lipoprotein, LDL: low density lipoprotein; FBS: fasting blood sugar; sys BP: systolic blood pressure; dya BP: diastolic blood pressure

**Patients’ lifestyles:** The mean disease duration was 42.7±60.6 months. Only 34.73% of lupus patients had high physical activity during the day (more than an hour/day physical activities for their job or more than 30 minute/day exercise program). In total, 24.7% of our patients followed a routine daily exercise program. The mean duration of activity in these patients was 2.6±2.37 h/day. 78 percent of the patients were taking some type of supplementary pills like omega 3, calcium-vitamin D, multivitamins.

**Metabolic syndrome and its components in patients and controls:** Twenty-seven (18%) lupus patients and 64 (29.1%) healthy controls had MetS according to ATPIII criteria. As shown in table-1, MetS was more prevalent in healthy controls than lupus patients. As demonstrated in table-1, the mean serum values of TG, FBS, and the average systolic and diastolic BP were significantly higher in lupus patients compared with healthy volunteers. However, waist circumference was significantly higher in healthy controls compared with lupus patients. HDL-cholesterol serum values did not show any significant difference between the two groups. The mean BMI and LDL-cholesterol were higher in healthy controls than patients were (table 2).

**Metabolic syndrome, disease related laboratory findings, medications and organ involvement in lupus: **There was no significant difference between cumulative prednisolone dosage in SLE patients with (58.8±45.4 mg/month) and without (43.9±35.1 mg/month) MetS (P=0.7, z=0.2). Moreover, the mean prednisolone dosage during the year prior to enrollment to the study was not different in patients with (17.9±25.6 g/year) and without (9.52±14.7 g/year) MetS (P=0.12, t=1.5). 

Proportions of SLE patients with and without MetS taking hydroxychloroquine was similar (77.8% vs/ 79.7% respectively, P=0.8). Similarly, the percentage of patients who received cytotoxics in patients with and without MetS was not statistically significant (18.5% vs/ 17.9% respectively, P=0.9). The mean duration of daily activity in patients with and without MetS was not significantly different (2.23±2.23 vs/ 2.23±2.14 hours/day, respectively, P=0.9). There was not any statistical difference between following items in lupus patients with and without MetS: serum levels of C3 (P=0.1), C4 (P=0.2), anti anti-DNA/R (p=0.8) as well as frequency of organ involvement (P=0.3). The mean age of lupus patients with MetS (33.9±9.7 year) was significantly higher than those without MetS (29.2±10 year) (P=0.009, t=2.6). However, age in controls with MetS (34.6±1.0 year) and without MetS (34.7±0.6 year) did not show any significant difference (P=0.4, t=0.7).

**Table 2:** Comparison of anthropomorphic factors other than MetS between individuals with and without MetS in lupus patients, controls and total participants

**Anthropomorphic variables &** **Metabolic syndrome status**	**Lupus patients**		**P-value**	**Healthy controls**		**P-value**	**Total participants**		**P-value**
	**Mets (+)** **Mean±SD**	**MetS (-)** **Mean±SD**		**MetS (+)** **Mean±SD**	**MetS (-)** **Mean±SD**		**Mets (+)** **Mean±SD**	**MetS (-)** **Mean±SD**	
LDL-cholesterol (mg/dl)	95.4±24.4	96.4±32.6	0.1, t=0.9	116.9±31.2	106.1±26.9	0.01, t=2.5	107.2±8.1	104.08±12.3	0.01, t=2.6
BMI (kg/m^2^)	25.3±4.5	24.3±4.3	0.2, t=1.1	30.6±5.5	26.6±5.1	0.001, z=4.6	27.1±1.9	26.2±2.4	0.001, z=3.2
Hip circumference (cm)	95.4±8.5	96.03±8.3	0.7, t=0.3	109.04±8.2	102.2±8.7	0.001, t=5.2	102.1±4.6	100.3±5.2	0.002, t=3.1

## Discussion

The results of this study indicate lower prevalence of MetS in SLE than healthy controls. MetS was not related to cumulative prednisolone dosage and duration of corticosteroid therapy.

 The average of BMI in lupus patients was lower than the healthy population despite glucocorticoid therapy. Frequency of MetS in lupus patients was 18% and in the control subjects was 29.1%.

Several studies on this topic found high frequency or higher prevalence of MetS in SLE than normal age and sex matched controls table 3 (3, 5-6, 9, 11-13, 15, 17-18, 25, 28). The main result of most of the aforementioned studies was that in lupus patients, MetS was more frequent than healthy volunteers. In our study, in contrast, MetS was less frequent than matched healthy controls. We also found the same results in RA patients in another study (29). Although different inclusion criteria for MetS may explain those discrepancies that were projected in table-3, we suppose that MetS criteria may not be applicable for diagnosis of MetS in SLE or RA. 

The difference may also be attributed to variations in disease characteristics, treatment methods and drug dosage in different studies. In particular, treatment with hydroxychloroquine, by its lipid lowering effect, may reduce lipid profile in lupus patients. Besides, periodic visits by giving cautions about hyperglycemia, hyperlipidemia and hypertension may reduce the risk of MetS in these patients. 

**Some other notable outcomes of the current research were as follows: **this study and our previous study on RA (30) showed high prevalence of MetS in the general population. Drugs such as hydroxychloroquine, cumulative dose of glucocorticoids and/or duration of glucocorticoid therapy showed no relationship to MetS development in patients. It was against the hypothesis that glucocorticoids may increase the risk of metabolic syndrome secondary to hyperglycemia and hypertriglyceridemia (30). Additionally, disease markers did not have any relationship to MetS development. Several studies were in line with our study on drug or disease activity influence on MetS development in lupus (3, 17-18, 25). 

Generally, lupus patients had statistically significant lower BMI, LDL and waist circumference than healthy matched individuals did. Lower BMI with the average of 24.56(±2.4) kg/m^2^shows an acceptable BMI for lupus patients despite of high dose of glucocorticoid therapy in most of them. 

Although we think that obesity is an important problem in our country, our patients were not overweight probably due to lupus-related cytokine over production, malnutrition and loss of appetite due to the disease nature or drug-related dyspepsia. As far as lifestyle habits were concerned, only 24% of patients followed a low fat low salt diet. In addition, 34% of patients had enough physical activities or were categorized as highly active. It expresses that our patients need to pay more attention to educational program for healthy lifestyle modification.

**Conclusion:** In aggregate, MetS showed higher frequency in apparently normal matched volunteers than lupus patients. Disease manifestations and drugs such as glucocorticoids or hydroxychloroquine had no relationship with MetS in lupus patients. BMI, LDL and waist circumference in lupus patients were significantly lower than in normal subjects. Most of the patients did not follow any healthy regimen or daily activity program.

## Notes

**Funding:** This article was extracted from the thesis prepared by Mina Khodabandeh to fulfill the requirements needed for earning the general practitioner’s degree. The Research Council of Mashhad University of Medical Sciences, Mashhad, Iran, is appreciated for financially supporting this study, grant number [89738].

## Citation:

Hatef-Fard M, Khodandeh M, Sahebari M, et al. Metabolic syndrome in lupus patients in northeast of Iran, and their lifestyle habits. Caspian J Intern Med 2016; 7(3): 195-200.
